# Supplementary figures and images for: Discriminative feature of cells characterizes cell populations of interest by a small subset of genes
Source: PLoS Comput Biol. 2021 Nov 19;17(11):e1009579. doi: 10.1371/journal.pcbi.1009579 (PMC8641884; doi:10.1371/journal.pcbi.1009579)

**Figure S1\_Fujii****a**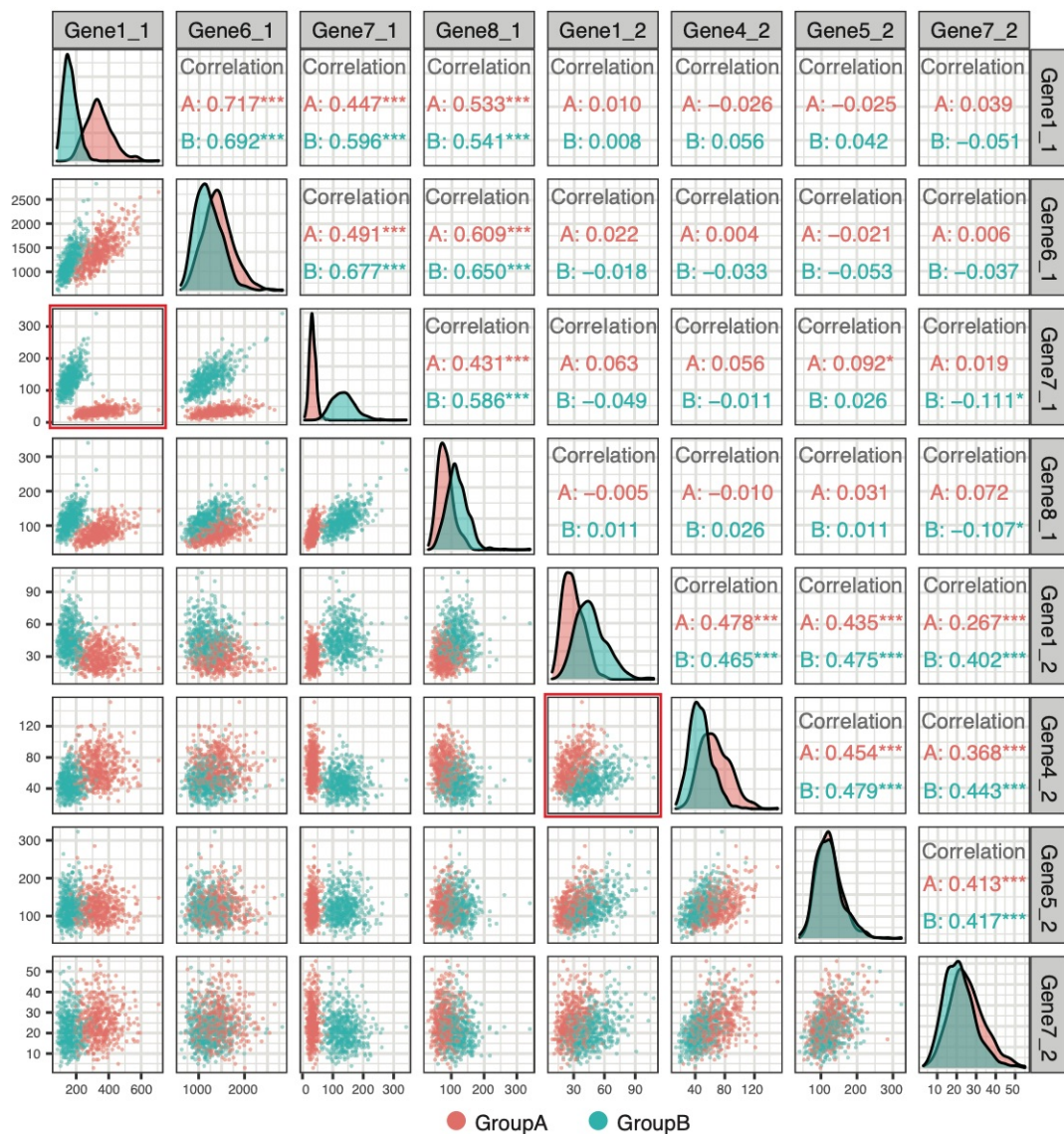**b**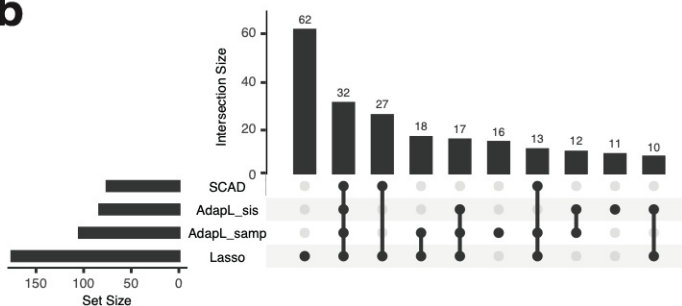**c**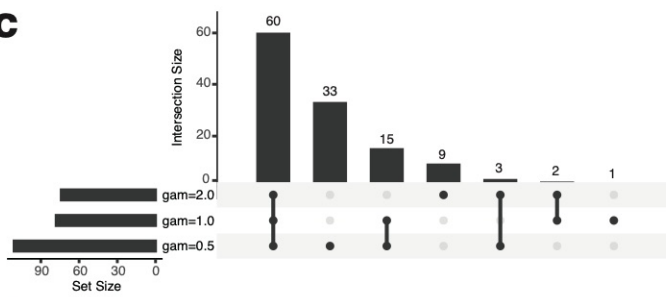**d**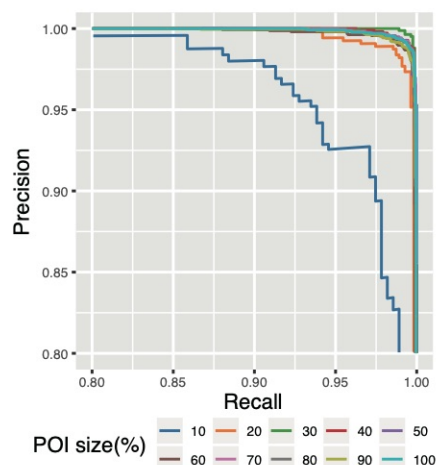

Supplement: S1 Fig — (a) Pairs of marker genes that are simulated by ESCO. The lower triangle shows the plot of each pair of variables; the diagonal elements show the distribution of each variable and the upper triangle shows the correlation coefficient within the cluster of each two variables. The highlighted pairs are prioritized in DFC selection. (b) UpSet plot to compare DFC sets extracted by each four methods (SCAD, SIS + adaptive LASSO, Sampling + adaptive LASSO and LASSO). Each columns represents number of genes that are shared by only the marked sets. (c) UpSet plot to compare DFC sets extracted by SIS + adaptive LASSO with γ = 0.5, 1, 2. (d) As the ratio of A to B increases, the performance of the generated model deteriorates. The PR curve when the sample size of POI is gradually decreased (100%, 90%,…, 10%). (PDF) [file pcbi.1009579.s001.pdf]

# Figure S4\_Fujii

**a**

## Strong feature

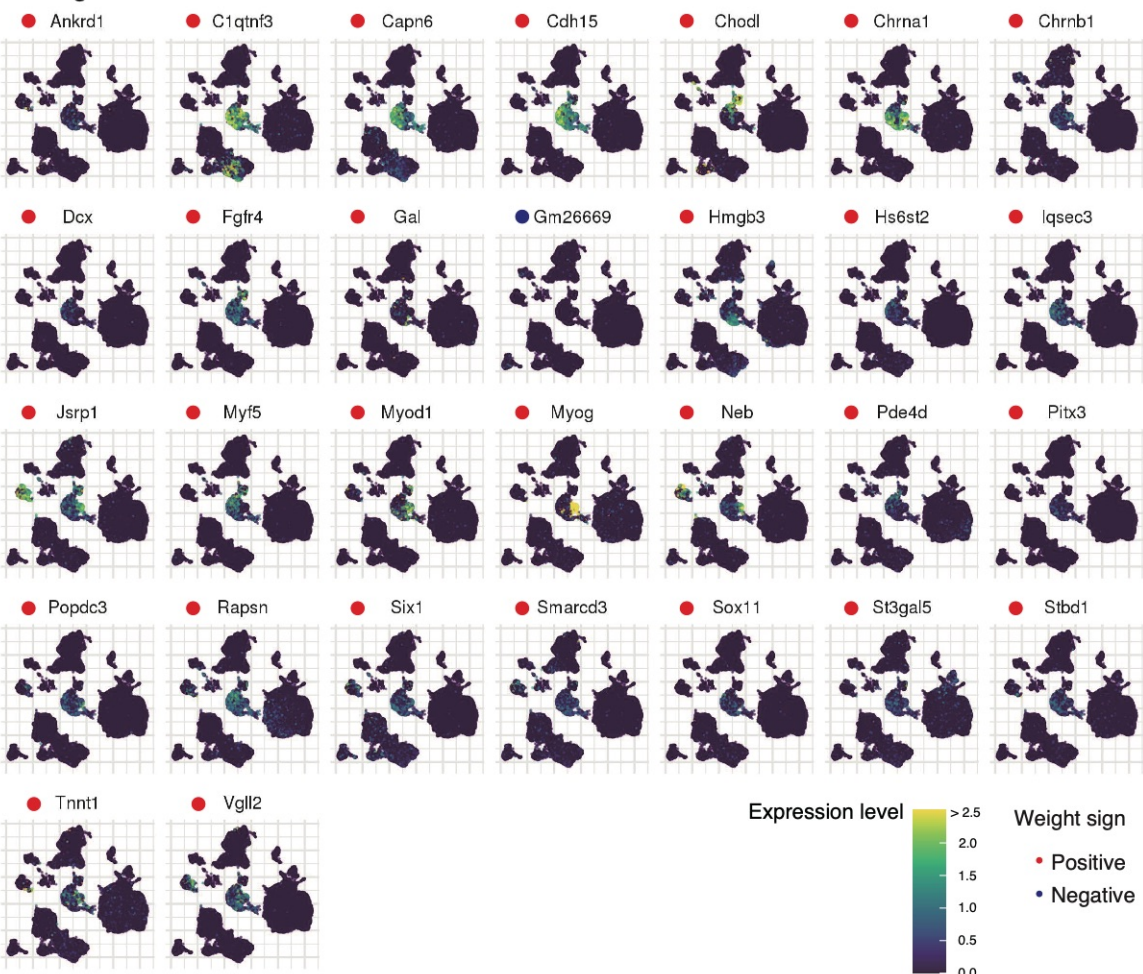

**b**

## Niche feature

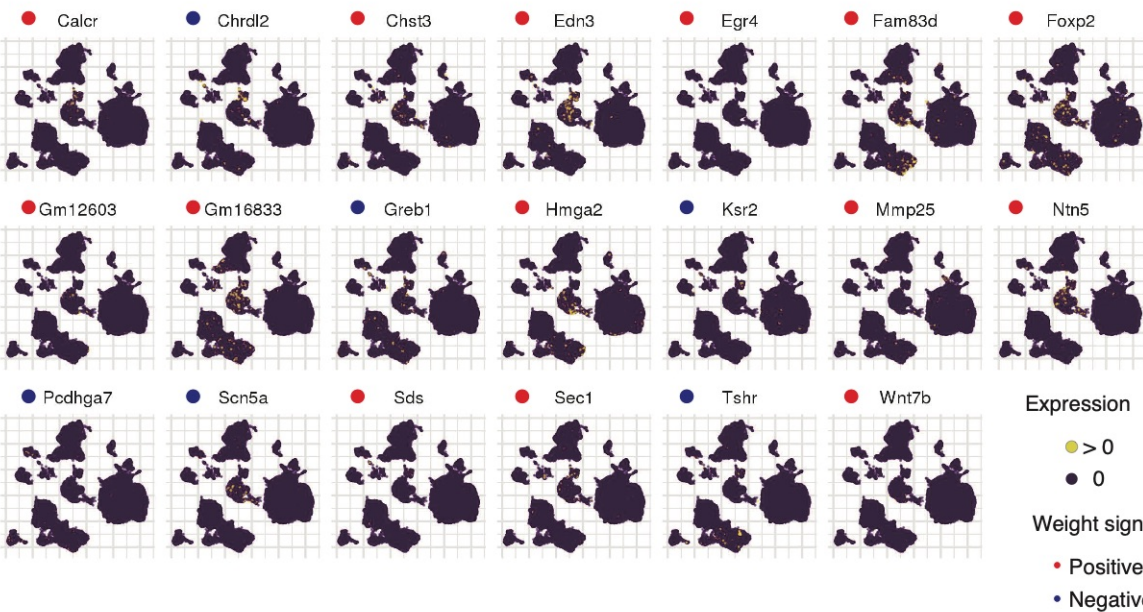

Supplement: S4 Fig — UMAP visualizations of (a) Strong and (b) Niche features. The markers colored in red/blue indicate the sign of the weight (coefficient) estimated by adaptive LASSO. (PDF) [file pcbi.1009579.s004.pdf]

# Figure S5\_Fujii

## Weak feature

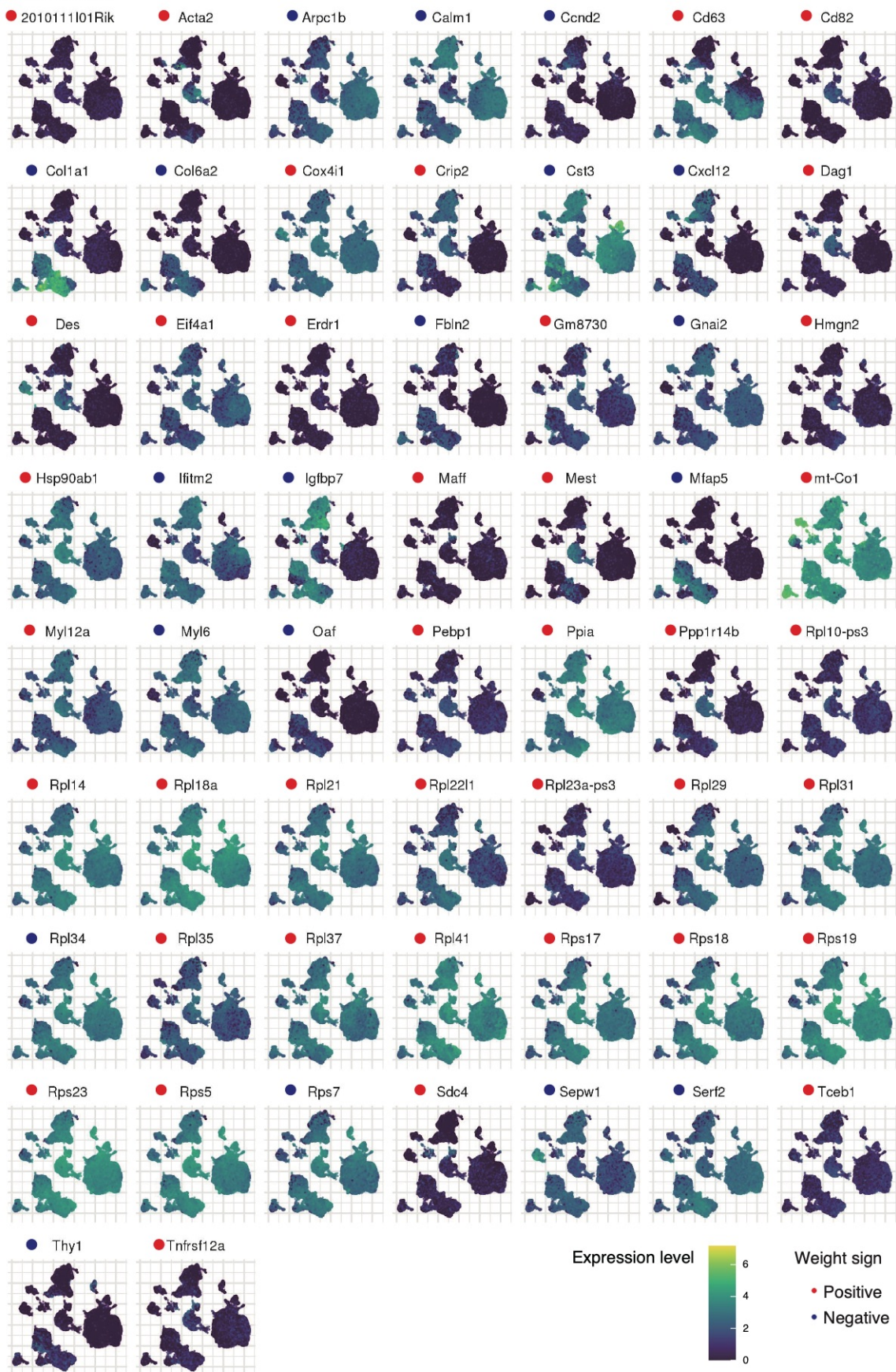

Supplement: S5 Fig — The markers colored in red/blue indicate the sign of the weight (coefficient) estimated by adaptive LASSO. (PDF) [file pcbi.1009579.s005.pdf]

**a**

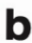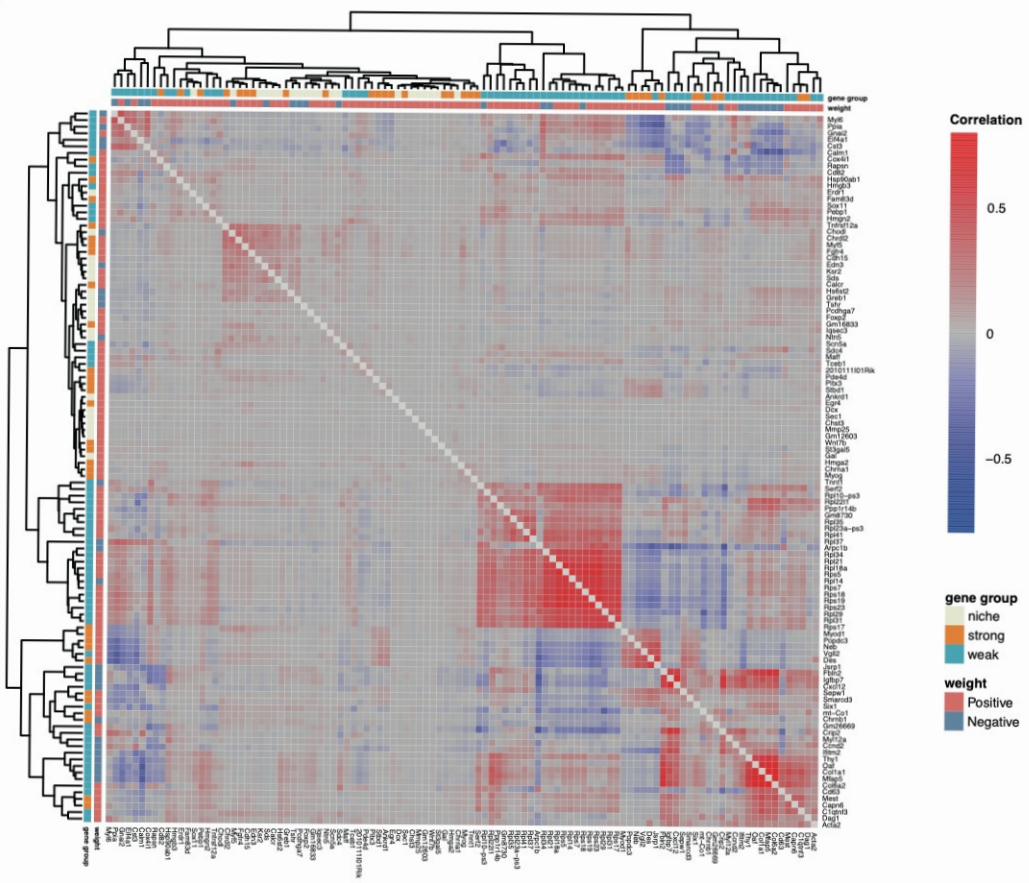

Supplement: S6 Fig — The matrixes in (a) POIs and in (b) Others are shown. Types of feature (Strong, Weak, or Niche) and the signs of LASSO weight are also indicated. (PDF) [file pcbi.1009579.s006.pdf]
