# Supplementary material for: Discriminative feature of cells characterizes cell populations of interest by a small subset of genes
Source: PLoS Comput Biol. 2021 Nov 19;17(11):e1009579. doi: 10.1371/journal.pcbi.1009579 (PMC8641884; doi:10.1371/journal.pcbi.1009579)

Figure S2_Fujii

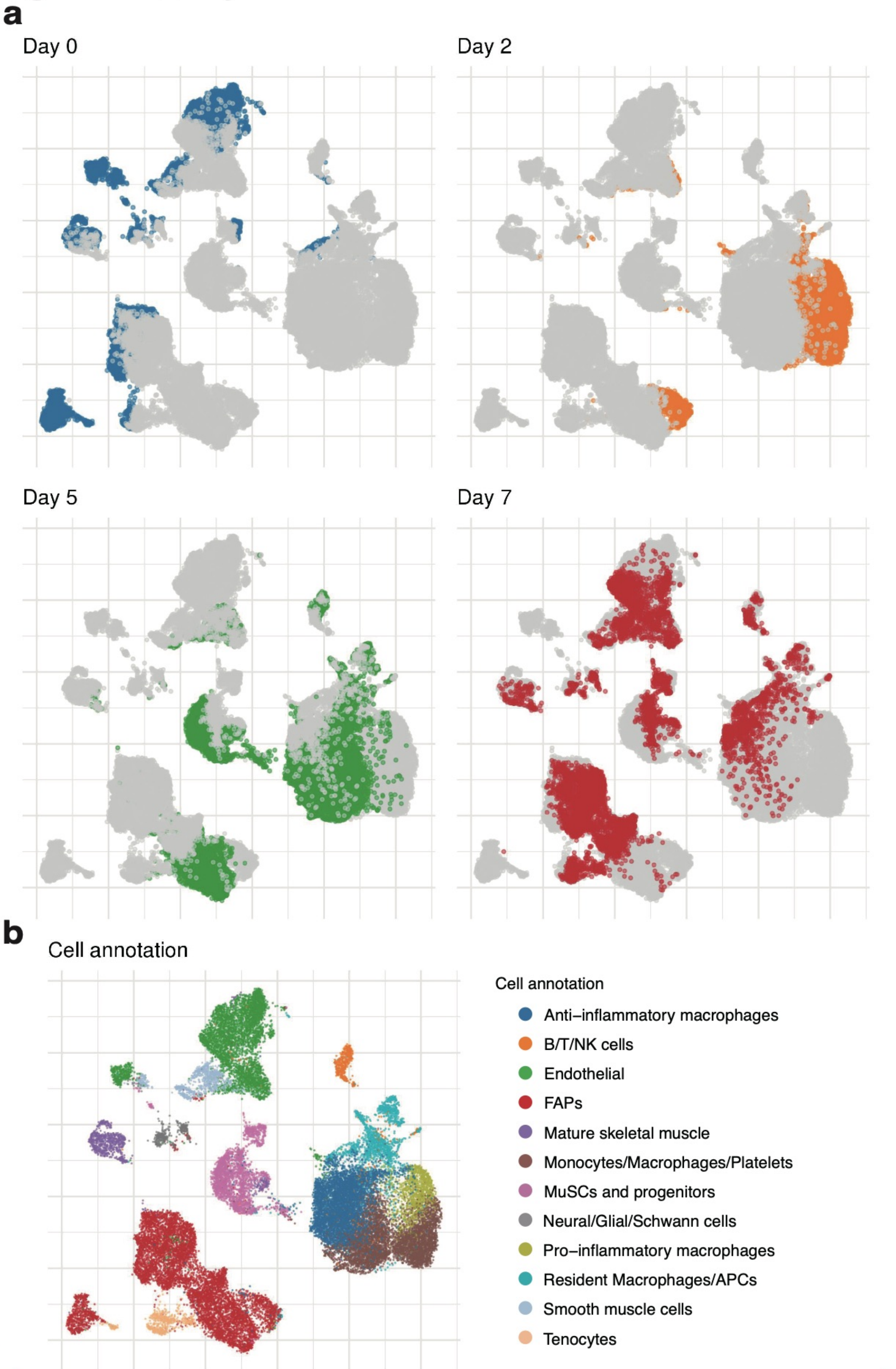

Supplement: S2 Fig — (a) Days after muscle injury. (b) Cell type annotations. (PDF) [file pcbi.1009579.s002.pdf]
