# Supplementary material for: Discriminative feature of cells characterizes cell populations of interest by a small subset of genes
Source: PLoS Comput Biol. 2021 Nov 19;17(11):e1009579. doi: 10.1371/journal.pcbi.1009579 (PMC8641884; doi:10.1371/journal.pcbi.1009579)

Figure S3_Fujii

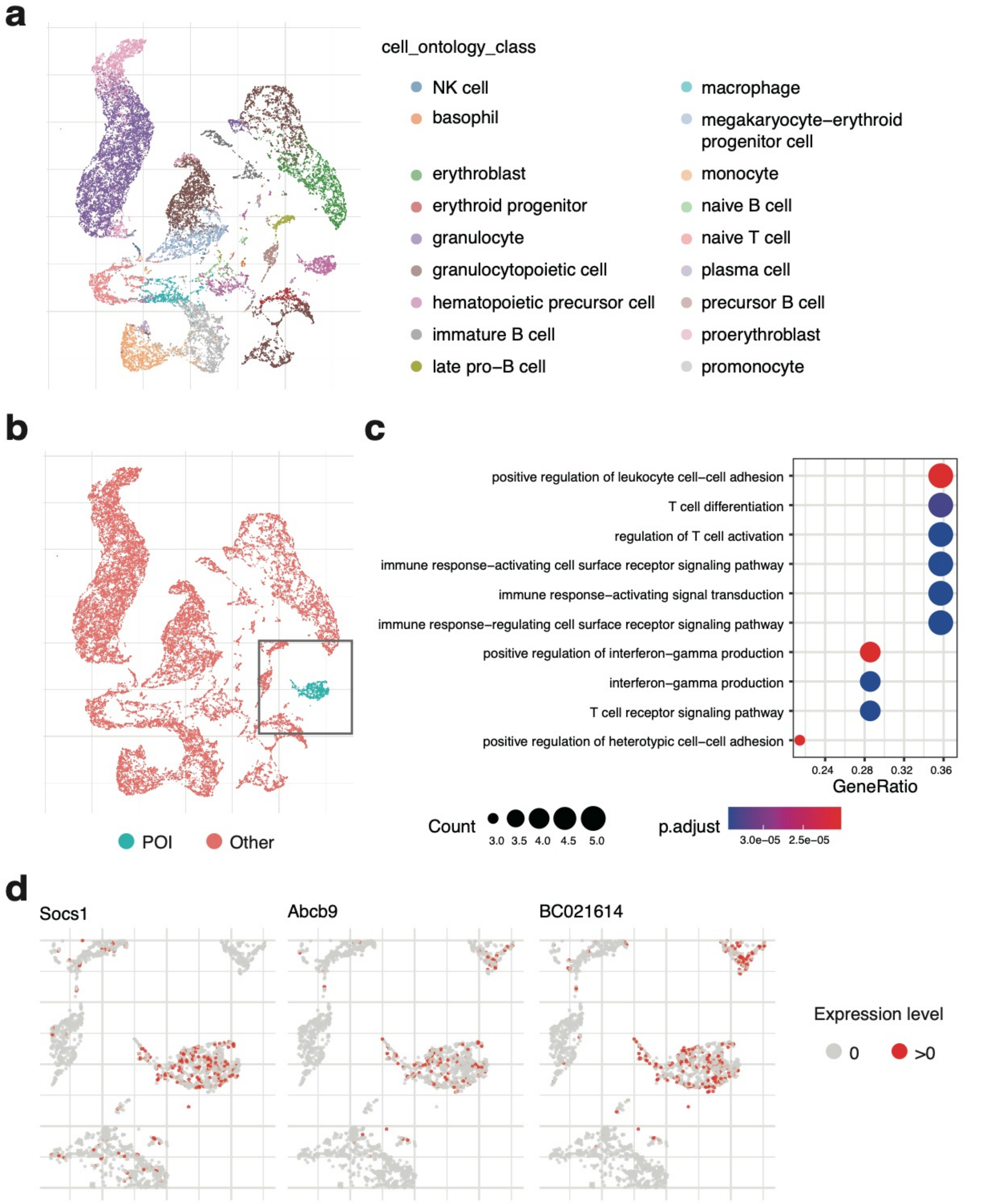

Supplement: S3 Fig — (a) The scRNA-seq data embedded into two-dimensional space with UMAP. (b) ​​”POI” and “Others” determined by the Louvain algorithm. POI corresponds to the cluster of hematopoietic precursor cells. (c) The Strong features contain many genes that are the markers of POI. The results of GO enrichment analysis for the Strong features. GOs are ordered by the contained proportion of Strong feature genes. (d) Zoom in on the area indicated in Fig S3b. Cells expressing Niche features (Calcr, Edn3, and Gm12603) are highlighted. (PDF) [file pcbi.1009579.s003.pdf]
