# Supplementary material for: Discriminative feature of cells characterizes cell populations of interest by a small subset of genes
Source: PLoS Comput Biol. 2021 Nov 19;17(11):e1009579. doi: 10.1371/journal.pcbi.1009579 (PMC8641884; doi:10.1371/journal.pcbi.1009579)

# Figure S8_Fujii

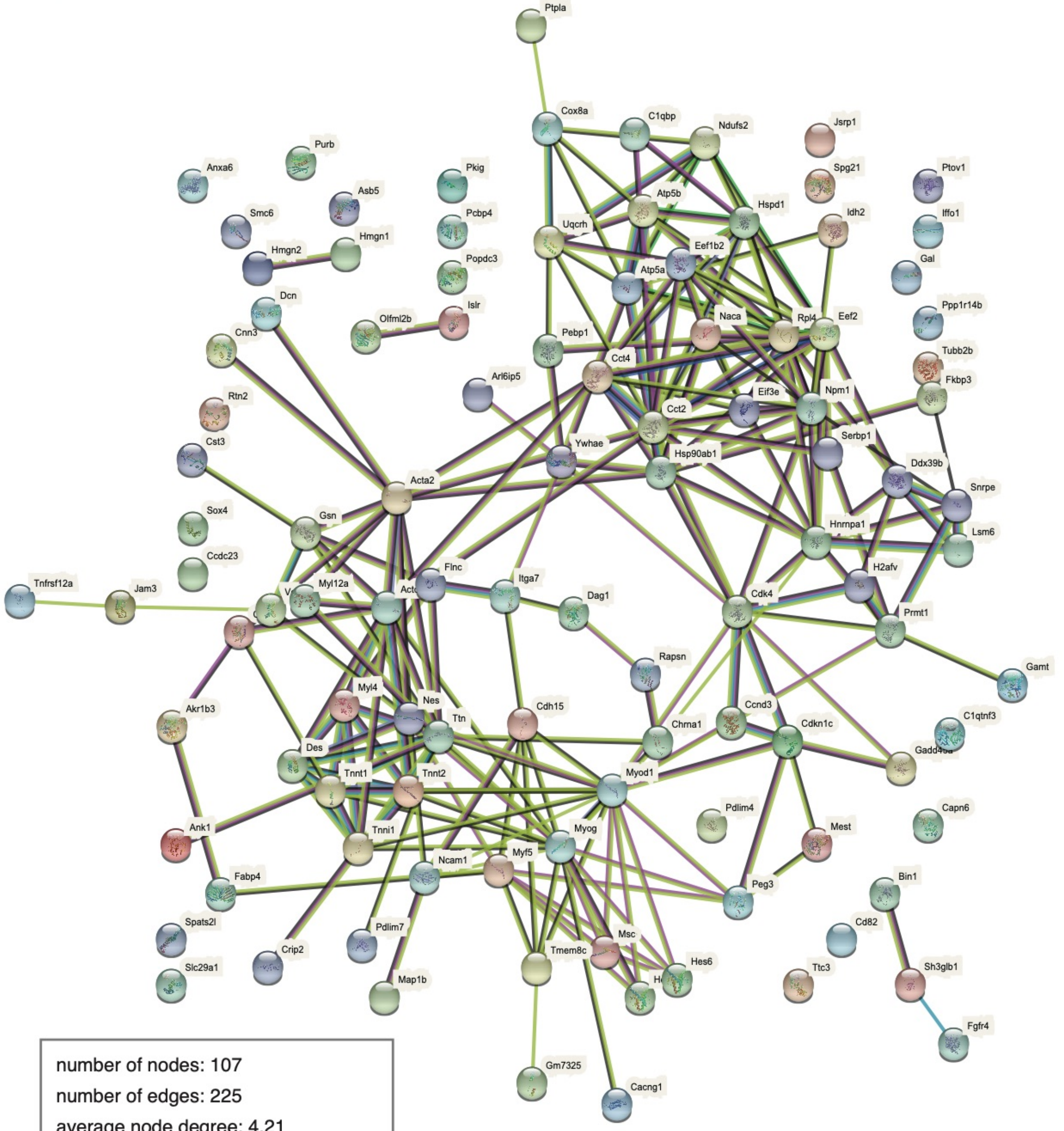


number of nodes: 107
number of edges: 225
average node degree: 4.21
avg. local clustering coefficient: 0.423
expected number of edges: 80
PPI enrichment p-value: < 1.0e-16

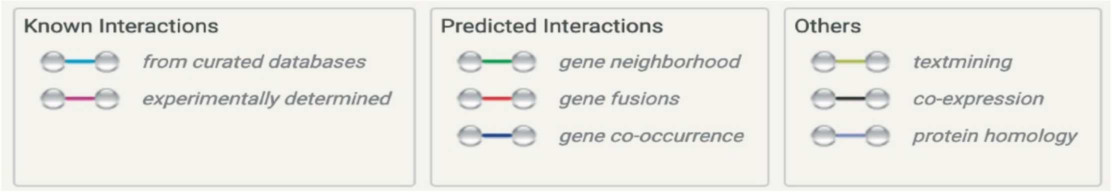

Supplement: S8 Fig — The functional associations of the top 108 genes in terms of P-value among the DEGs annotated by STRING. (PDF) [file pcbi.1009579.s008.pdf]
