# Supplementary material for: Discriminative feature of cells characterizes cell populations of interest by a small subset of genes
Source: PLoS Comput Biol. 2021 Nov 19;17(11):e1009579. doi: 10.1371/journal.pcbi.1009579 (PMC8641884; doi:10.1371/journal.pcbi.1009579)

Figure S9_Fujii

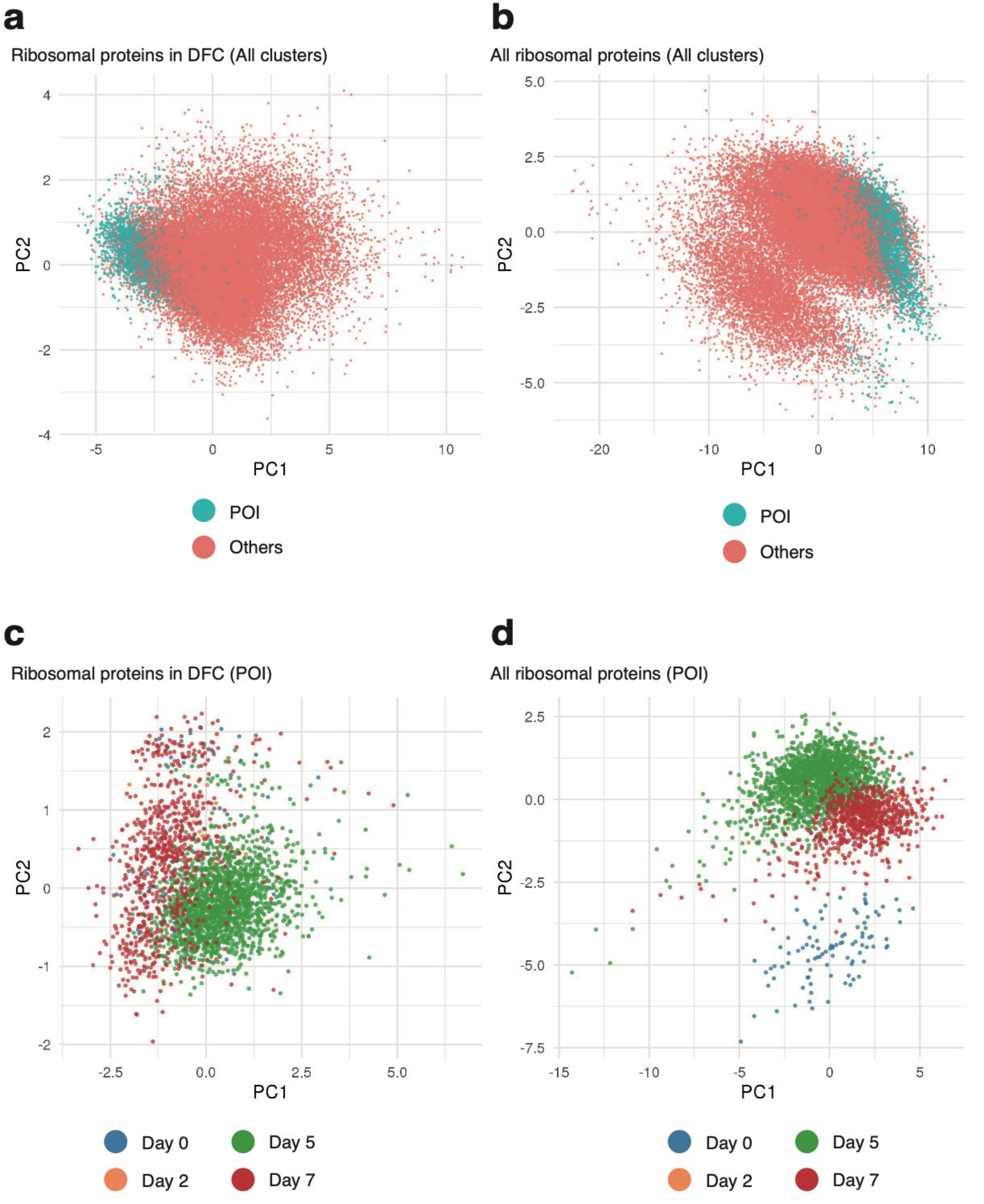

Supplement: S9 Fig — Scatter plot showing the results of PCA performed using (a) ribosomal protein-coding genes in DFC and (b) all ribosomal protein-coding genes. (c) PCA performed in the POI using genes in (a), and in all cells (POI and others) using genes in (b). (PDF) [file pcbi.1009579.s009.pdf]
