# Supplementary material for: Discriminative feature of cells characterizes cell populations of interest by a small subset of genes
Source: PLoS Comput Biol. 2021 Nov 19;17(11):e1009579. doi: 10.1371/journal.pcbi.1009579 (PMC8641884; doi:10.1371/journal.pcbi.1009579)
Supplement: Attachment — Submitted filename: Response_to_Reviewers.docx [file pcbi.1009579.s012.docx]

Reviewer #1: The authors have proposed using the Elastic Net package in R to address the limitation of differentially expressed gene identification. The idea looks legitimate. However, the technical contribution to methodology could be limited. There are several key questions which have to be addressed to fit into the preference of computational biology, on top of bioinformatics.

Our main goal in this manuscript is to provide practical directions to apply discriminative methods for profiling unknown cell populations derived from single-cell data instead of DEG, rather than to propose discriminative or DEG methods themselves. There are several reports describing the use of discriminative methods to analyze scRNA-seq data for the purpose of discriminating between cancer and normal cells. However, there has been no discussion on how to link the characteristics of gene sets selected by discriminative methods (characteristics of gene expression prioritized by discriminative performance and sparsity constraints) with biological interpretation. Here, we proposed adaptive LASSO as one form of implementation of the concept of DFC, but by incorporating this referee’s proposal, we were able to deepen the discussion on the choice of methods other than adaptive LASSO and the biological interpretation. We appreciate this valuable advice.

Please see the point-by-point responses written in blue below.

1. It is straightforward to expect that the adaptive LASSO can always identify a small set of key genes for cell type / population identification. Therefore, the DFC set looks smaller than the DEG. I was wondering what are the biological significance of that DFC set? Are those genes only used to differentiate cell populations?

The point raised is of great importance and we consider it as follows. DFC is a discriminative feature and has the ability to identify cell populations. Genes extracted by DFC could be classified into three groups by focusing on the specificity of expression:

(1) the specific marker genes of the POI as extracted by DEGs;

(2) genes that characterize subpopulations in the POI; and

(3) genes that are characterized by expression patterns of multiple genes.

In summary, we believe that DFC is significant in terms of its ability to extract subpopulations and expression networks that define populations. To reflect this in the text, we have added the following to the discussion:

“DFC has the potential to identify subpopulations within the population of interest and extract gene networks that regulate these subpopulations, in addition to the conventional identification of expression markers in cell populations, and is expected to significantly advance our understanding of cell populations from gene expression analysis.”

What are the implications behind the DFC set. Is the DFC set reflecting / overlapping with the "essential genes" ?

On the basis of this comment, we examined the overlap with essential genes. We used the definition given by Dickinson as the list of essential genes. The essential genes in the DFC set were comparable to the random chance, i.e. the result did not show any tendency for enrichment. In addition, the DFC set showed unique genes that could define a cell population, with the suggestion of enrichment of protein–protein interaction, as shown by the STRING analysis. These findings suggest that DFC could extract a unique feature that is independent from essential genes. We thus revised the text as follows:

“We also examined how many DFC belong to Essential genes, which are defined by Dickinson et al. as genes essential for survival. According to this definition, 5280 genes were essential genes, and 26.58% of DFC were essential genes in the data used in this study. In contrast, the number of essential genes in DFC was 32 (32/108 ≈ 0.30). Therefore, there was no tendency for essential genes to be selected as DFC. Genes that are essential for survival do not need to be present as a characteristic of a particular POI.”

2. The technical contribution to the methodology looks limited as it is only based on the off-the-book-shelf adaptive LASSO method from the well-known Elastic Net package in R. Comparisons to other feature selection or model regularization methods in machine learning could be beneficial.

We adopted adaptive LASSO as the most suitable sparse regression method for the purpose of DFC because it has shown high performance in recent reviews and has consistency in interpretation. However, as pointed out by this referee, our proposal would be more convincing if we had the results of a comparative study of similar methods. In this study, we compared three methods: SCAD, LASSO, and SIS-Adaptive LASSO. As described in the main text, we used scRNA-seq data of mouse tibialis anterior to extract DFC with MuSCs and their progenitors as the POI.

In either method, the number of genes was around 100, and the levels of discriminative performance itself were comparable. The most striking feature was the significant reduction in computational time due to the adoption of SIS. We would like to thank the referee for their suggestion.

For the DFC set extracted by each method, we created UpSet plots (Fig. S1c) and analyzed the association between genes using the STRING database (Table S1). The UpSet plots show that LASSO extracted a large number of genes and contained many specific genes, and that there were many genes in common between Sampling + adaptive LASSO and SIS + adaptive LASSO. In addition, Sampling + adaptive LASSO showed the highest average number of edges (associations), while conversely this number was relatively low in SCAD. These results suggest that adaptive LASSO can extract a more biologically meaningful gene set in terms of the gene associations (e.g., co-expression, PPIs, pathways) than other discriminative methods.

The following was added to the text.

“Table S1 summarizes the averages of the computational time and the number of extracted genes for DFC and DEG extraction for each method. In addition to the Sampling + adaptive LASSO–logistic regression and Wald test (DESeq2) used in this study, the results of the same analysis using five other methods are also shown. For DFC extraction, we repeated the analyses ten times for averaging the effect of subsampling and cross-validation. For the other option to detect DEGs, limma[5] is known to be effective for DE analysis in single-cell RNA-seq data[10], the large number of genes was extracted as in DESeq2. For the DFC set extracted by each method, we created UpSet plots (Fig. S1c) and analyzed the association between genes using the STRING database (Table S1). The UpSet plots show that LASSO extracted a large number of genes and contained many specific genes, and that there were many genes in common between Sampling + adaptive LASSO and SIS + adaptive LASSO. In addition, Sampling + adaptive LASSO showed the highest average number of edges (associations), while conversely this number was relatively low in SCAD. These results suggest that adaptive LASSO can extract a more biologically meaningful gene set in terms of the gene associations (e.g., co-expression, PPIs, pathways) than other discriminative methods.

In the extraction of DFC, the performance of SIS + adaptive LASSO was better than that of Sampling + adaptive LASSO in terms of computational time and the c.v. error. Therefore, the use of SIS may be a promising option to reduce computational costs. However, in this study, we decided to use all genes by sampling cells to search a wide range of feature genes.”

3. The underlying pathways behind those DFC genes could be interesting.

Following the referee’s suggestion, we added analysis of the DFC of MuSCs and their progenitors using Reactome, which is an extensive database of pathways in mice. In addition to multiple pathways related to ribosomal proteins (such as rRNA processing and translation), there are pathways related to skeletal myogenesis, such as myogenesis. Myogenesis is consistent with the properties of MuSCs and their progenitors, which are the POI, and the activation of transcription is also discussed as being related to skeletal myogenesis, which is also consistent with the properties of the POI. Therefore, it is suggested that pathway analysis may also be useful in determining the nature of the POI.

As such, we revised the text as shown below.

“We performed pathway analysis with the Reactome database using all DFC (Table S2). We found pathways enriched in ribosomal proteins such as rRNA processing and translation, and pathways enriched in skeletal muscle marker genes such as myogenesis. These results are consistent with the results of DFC set analysis by STRING (Fig. 3j) and GO enrichment analysis using the Strong feature. Therefore, pathway analysis is also useful for investigating the nature of DFC or POI.”

4. The concept of DEG has been well-established in the past years. It is therefore important to tell the difference between DFC and DEG. The overlapping and non-overlapping genes between DFC and DEG should be analysed further to reveal its biological difference / similarity.

We agree with this comment that it is important to clarify the relationship between overlapping and non-overlapping genes between DFC and DEG. In this regard, our interpretation is as follows. DFC genes that do not overlap with DEGs are often found in niche features, which have low expression and exhibit characteristics that characterize subpopulations in POIs. The niche feature is characterized by its low expression level, which characterizes the subpopulation in the POI. Therefore, it cannot be tested due to its low expression level and tends not to be extracted by DEG-based analysis. In contrast, the overlapping genes can be divided into two categories: Strong features, which are distinct marker genes; and Weak features, which are characterized by combinations.

In principle, DFC was designed to reduce the number of genes to detect since DEG has been faced to detect numerous genes.To reflect the discussion you pointed out, we have revised the manuscript with the following text.

““DFC genes that do not overlap with DEGs are often found in niche features, because it cannot be tested due to its low expression level and tends not to be extracted by DEG-based analysis. In contrast, the overlapping genes can be divided into two categories: Strong feature and Weak feature. In principle, DFC was designed to reduce the number of genes to detect since DEG has been faced to detect numerous genes.”

5. The running time and computing environment should be reported as gene expression data can be very big in scale.

We agree that a comparison of the computational time is important in comparing methods. Running time is summarized in Table S1. All calculations in this study were performed in the following environment.

- CPU: Intel Xeon Skylake-SP (2.3 GHz, 18 core) × 2 CPU

- Memory: 384 GB

We revised the text as shown below.

“All calculations in this study were performed under the following environment.

- CPU: Intel Xeon Skylake-SP (2.3GHz, 18core) x 2CPU

- Memory: 384GiB”

Reviewer #2: In this manuscript, authors proposed a new concept of discriminative feature of cells (DFC), which was an alternative to the DEG-based approach that uses lists of genes with differences in expression. DFC was a discriminative approach implemented using adaptive LASSO logistic regression. The results revealed that DFC well captured cell-type-specific markers, specific gene expression patterns, and subcategories of the cell population. Some comments are given below.

We appreciate your comments and believe that our manuscript was improved by incorporating your suggestions.

Please see the point-by-point responses below.

1. For section “Adaptive LASSO–logistic regression” starting from line 380, the adaptive Lasso used was a little different from the method in reference [13]. For computations of adaptive LASSO, the gamma parameter in the weights or penalty factor was directly set to be 1. In the reference, the optimal pair of gamma and lambda parameters was obtained by cross-validation. It is suggested to explain why gamma was directly set to be 1.

Thank you for the comment about this point. We did not optimize the hyperparameter *γ*.

In reference [14], cross validation was done for *γ* = 0.5, 1, 2. We also set the values of *γ* = 0.5, 1, 2 and evaluated the extracted variables. There was no significant difference in the cross validation error, but *γ* = 1 showed a slightly smaller value. Also, as the *γ* increased, the number of selected variables decreased, but the variables selected were consistent. Therefore, we decided to use *γ* = 1 in this manuscript.

We added the following to the text.

"The parameter *γ* for adaptive LASSO, which controls the size of the ridge penalty, was evaluated using the faster SIS + adaptive LASSO. The number of selected genes decreased as the *γ* increased, but the selected genes showed consistency (Fig. S1c). In addition, the cross validation error was minimized at *γ* = 1. Therefore, we decided to use *γ*  = 1 as the fixed value."

2. Ridge regression estimates were used in the first step with 30% subsamples of cells from each cluster. Please give more details about the sample size and the number of variables involved. For ultrahigh dimensional statistical models, (I)SIS is suggested. And based on the code available on Github, Ridge regression estimates were chosen by cross-validation (cv.glmnet), which is not shown in the manuscript. It is suggested to give more details of the implemented adaptive LASSO.

As indicated, we subsampled 30% of the cells from each Louvain cluster and performed ridge regression. The total number of cells after subsampling was 10,324. Genes with fewer than 10 cells expressed in the subsampled state were filtered out, and 16,351 genes were used as variables.

We thus revised the text as follows:

“We obtained 30% subsamples of cells from each cluster (16,351 cells in total ).”

By using SIS, we were able to reduce the computation time significantly. The results are summarized in Table S1. When performing SIS, the number of genes after screening was set to 824 genes. (Set based on SIS:::calculate.nnis, n_new = floor(n/(4 * log(n)))) In comparison with sampling + adaptive LASSO, a high percentage of 61/86 genes were duplicated. 11/86 genes corresponded to niche, although it was expected that niche features would be difficult to detect. ‘Niche feature detection', one of the features of DFC, was not impaired by the use of SIS.

Please see the comment for referee#1-2 for the detail.

As pointed out, the penalty for adaptive LASSO is based on the partial regression coefficient estimated by ridge regression. We have thus added the following to the Method section Adaptive LASSO-logistic regression.

We thus revised the text as follows:

“These ridge (first step) and LASSO (second step) regressions in the adaptive LASSO were performed by setting the hyperparameter α in the glmnet::cv.glmnet function to 0 or 1, respectively. The sparsity parameter λ was determined by 10-fold cross-validation (cv.glmnet) of binomial deviance. ”

3. Since logistic regression was involved, it is suggested to consider the impact of imbalanced binary response data.

We agree with this comment. The sample size ratio of POI to Others was 11.5, reflecting imbalanced data. We verified the deterioration in accuracy of the discriminative model caused by this imbalance.

When using actual data from mouse skeletal muscle in this study, the AUC of the precision–recall curve was ~1, indicating that an appropriate model could be created (i.e., this problem was not seen in this case). This can be attributed to the fact that we are comparing well-separated populations.

In addition, we examined the effect of increasing the imbalance. We subsampled the POI size to 10%, 20%, ..., 100% and trained the discriminative model (DFC extraction) using SIS + adaptive LASSO–logistic regression. Even with a 10% subsample, the AUC was about 0.98, indicating that it was possible to create a model with high performance. However, as the difference between the POI and Others increased, the performance tended to deteriorate. In addition, this property is not related to regularization, but to the problem of logistic regression. Moreover, the decrease in the performance of the model is not limited to the effect of imbalance, but can also be attributed to the decrease in sample size.

We thus revised the text as follows

“The sample size ratio of POI to Other is 11.5, reflecting imbalanced data. This imbalance may cause a decrease in the performance of the discriminative model. We therefore evaluated the AUC of the precision–recall (PR) curve. The AUC was ~1 (Fig. S1d), indicating that an appropriate model for discrimination had been created. We further examined the effect of increasing the imbalance between the sizes of POI and Other by training the discriminative model (extraction of DFC) using SIS + adaptive LASSO–logistic regression with a subsample of gradually decreasing POI size (Fig. S1d). Even with a 10% subsample, the AUC was about 0.98, indicating that the model has sufficient ability to discriminate between groups. This can be attributed to the fact that we selected well-separable populations in this case (comparison after clustering). In line with the principles of logistic regression analysis for imbalanced data, it is worth checking the predictive performance using PR curves to be aware of any problem of overfitting. Such an overfitted model would overlook the characteristics of the POI.”

4. It is suggested to compare adaptive LASSO with other variable selection methods, e.g. SCAD.

We agree with this comment, as also described in #1-2: We compared SCAD, LASSO, and SIS + adaptive LASSO with three similar methods (Table S1). We found differences in computational time, cross-validation error, and number of genes. Please see the comments above in response to #1-2 for details as well.

5. It is suggested to demonstrate the computation efficiency of DFC methods. For example, the computation time comparison between DEG and DFC methods.

We agree with this suggestion. We have also received a similar suggestion in comment #1-5. We demonstrated the the computation efficiency of DFC methods in TableS1, which shows the computational times for four DFC extraction methods and three DEG extraction methods. By using limma, which is considered to be suitable for DE analysis of single-cell data, it was possible to obtain DEGs very rapidly compared with the case with DFC. Please see the comments above in response to #1-5 for details as well.

6. It is suggested to do another real data analysis to validate the results for DFC.

Thank you for this suggestion. By adding the results of analysis of another data set, it is possible to show the wide applicability of DFC. Therefore, as per the process described in the original manuscript, we selected and analyzed the data with the aim of interpreting the POI using DFC.

The scRNA-seq data of mouse bone marrow in Tabula Muris were obtained and analyzed by setting the hematopoietic precursor cell population as the POI (target). Through analysis using these data, we confirmed that it is possible to use DFC to interpret the POI in different data as well as the results.

We thus revised the text as follows:

“To validate the method with other data, we obtained the data of bone marrow , a tissue composed of relatively similar cells with the same lineage, as most of the cells in the bone marrow are differentiated from hematopoietic stem cells. Therefore, we thought that DFC could provide a more detailed evaluation. After visualization by UMAP and clustering by the Louvain algorithm, the hematopoietic precursor cell population was set as the POI (target) for analysis (Fig. S3a,b). 31 genes were extracted, of which 14 were strong features, 14 were weak features, and 3 were niche features. Next, we attempted DE analysis by limma, and found 877 DEGs, of which two genes overlapped with DFC. Compared to the analysis of muscle tissue, the number of DEGs is small, but this may be due to the similarity of the cells that make up Bone marrow. Furthermore, these extracted genes were analyzed in detail by GO enrichment analysis. The GO enrichment analysis using strong features showed accumulation of myeloid cell differentiation-related terms such as interferon gamma production and T cell differentiation (Fig. S3c). This result also suggests that POI is a population of hematopoietic progenitors related to Lymphoid. In addition, three genes were classified as niche features, and Socs1 was included as a characteristic gene (Fig. S3d). Socs1 has been reported to be a negative regulator of the JAK-STAT pathway, which is involved in the regulation of myeloid cell differentiation and proliferation. It was thus suggested that Socs1 is a factor that characterizes a small subpopulation with suppressed differentiation and proliferation. ”

7. For the code on Github, string.nb.html is not displayed correctly.

We apologize for this error. We uploaded the correct file.

Reviewer #3: This article develops a new method to identify cell-type-specific genes based on single-cell RNA sequencing (scRNA-seq) data. The basic idea is to fit a classification model between the population of interest and other groups, and the identification of marker genes is viewed as a variable selection problem. In this article, the authors promoted the use of a logistic regression model with adaptive Lasso penalty, and genes with nonzero regression coefficients are defined to be discriminative features of cells (DFC).

Overall, this article introduces a tool that could be useful in practice, but I would hope that the authors can address the issues I list below.

We appreciate the positive comments by reviewer#3 on our manuscript. We have revised the manuscripts following his/her suggestion. We believe the current manuscript should address the points and point by point responses were shown below to each point.

1. The authors should have provided a more comprehensive review of existing works. The use of discriminative models for differential expression analysis is not completely new. For example, [1] fits a logistic regression to predict cell membership, although their predictors are transcripts instead of genes. The authors need to explain the overlap with prior art and highlight the novelty.

In this study, we show that the genes extracted by adaptive LASSO–logistic regression have biological significance as characteristics of the cell population to be analyzed.

It is true that there are already several attempts to apply discriminative methods such as support vector machine, random forest and sparse logistic regression to single cell RNA-seq data as we described in the Discussion in the initial manuscript. Therefore, we believe that it is novel to propose an analytical method focusing on individual extracted genes.

Thus, we also move the description of Ntranos’s work to the introduction part to emphasize the main focus of our work as below:

“Ntranos et al. incorporated logistic regression to detect transcription isoform changes from scRNA-seq data. We further pursue the applicability of a discriminative approach for characterizing cell groups, especially for tissue scRNA-seq data in which gene expression levels are correlated and there are heterogeneous subpopulations within a specified POI. ”

2. The proposed method was mainly compared with DESeq2. However, DESeq2 was originally designed for bulk RNA-seq data, and scRNA-seq data have certain characteristics that may harm the performance of these methods. This phenomenon was explained in [2] in more details. Therefore, the authors may want to consider some methods that are tailored for scRNA-seq, for example the ones listed in Table 1 of [2].

DESeq2 has sometimes been diverted to the analysis of single-cell data, but as the reviewer pointed out, it was originally a tool for the analysis of bulk RNA-seq data. Therefore, as suggested, we performed DE analysis using tools other than DESeq2, which is limma in this revised version of the manuscript. The results are shown in Table S1. limma-voom and limma-trend could detect DEGs very rapidly, but the number of extracted genes was extremely large, which is similar to DESeq2.

We thus revised the text as also shown in the response to #1-2.

3. The simulation of synthetic data can be made more realistic, as scRNA-seq data in real world contain more noise than the simple normal distribution used in the article. For example, the drop-out effect is one characteristic of scRNA-seq that should not be ignored. The authors may consider existing simulators such as Splatter [3] and ESCO [4].

The data used for Fig. 1 were generated using a simple normal distribution. While these data are easy to control, as pointed out, they differ from actual data. Therefore, we decided to follow the suggestion and use ESCO, a simulator that takes drop-out into account, to generate the simulated data and extract the genes using DFC. Through the simulation by ESCO, we can show that gene pairs with the same relationship as in Fig. 1b-d are preferentially extracted.

We revised the text to refrect this:

“We also investigated the nature of DFC using simulated data that more closely resemble real scRNA-seq data. We used ESCO, a simulator that takes drop-out into account, to generate the data. We designed the data with two cell populations and 15 genes. The two cell populations each have two marker genes. Two sets were generated and combined to create a data set containing gene sets derived from the two gene correlation networks (GCN) (500 cells for groupA and 493 cells for groupB) (Fig. S1a). Gene1_1, Gene6_1, Gene7_1, and Gene8_1 were marker genes generated from GCN1, while Gene1_2, Gene4_2, Gene5_2, and Gene7_2 were ones generated from GCN2. The correlation between marker genes generated from different GCNs is low. Using these data, we extracted the DFC for Group1. In this case, we controlled the sparsity (λ) and limited the number of variables to be extracted to four. As a result, Gene1_1, Gene6_1, Gene1_2, and Gene4_2 were extracted. From the scatter plot, we can see that the pairs of Gene1_1 and Gene6_1, and Gene1_2 and Gene4_2 are highly correlated within the class and have high separability. Therefore, through the simulation by ESCO, we can show that the gene pairs with the same relationship as in Fig. 1b-d are preferentially extracted.”

4. Minor comment: the text "in principle the P-value decreases with increasing sample size" in line 79-80 is not very accurate. Under the null hypothesis (no difference), P-value follows a uniform distribution, so it does not decrease to zero as sample size increases. The actual meaning is that a larger sample size can detect smaller and smaller differences, so with a fixed difference, a larger sample size usually gives smaller P-values.

As the reviewer pointed out, the expression was inappropriate regarding the relationship between p-value and sample size. We have thus corrected the relevant part as follows:

“a larger sample size can detect increasingly small differences, with a fixed difference, a larger sample size usually gives smaller P-values, resulting in the unnecessary expansion of candidate genes ”

5. Minor comment: in Figure 1d and 1g, what are the actual lambda values? Does cross validation correctly select the sparse model?

Since fig1d and fig1g show the relationship between *λ* and the partial regression coefficients, we did not select a specific *λ* value. For the bounds shown in fig1c and fig1f, we set 0.026 and 0.227 as *λ* , respectively, and calculated the parameters. This value is not the obtained value from cross-validation, but since we are discussing gene prioritization here, we do not aim to retrieve the optimal set of variables there. Therefore, we used the above value for *λ* , which is the value that gives the minimum *λ* under the condition that two variables are selected. In contrast, the coefficients of the ridge regression used for the penalty were obtained by cross-validation.

We thus revised the text as follows:

“To calculate the slope and intercept of the decision boundary in Fig. 1c, we used the minimum value of *λ* that gives two variables are selected. ”

“The slope and intercept of the decision boundary were obtained in the same way as in Fig. 1c. ”
