# Supplementary material for: Discriminative feature of cells characterizes cell populations of interest by a small subset of genes
Source: PLoS Comput Biol. 2021 Nov 19;17(11):e1009579. doi: 10.1371/journal.pcbi.1009579 (PMC8641884; doi:10.1371/journal.pcbi.1009579)
Supplement: Attachment — Submitted filename: Responce_to_Reviewers_v2.docx [file pcbi.1009579.s013.docx]

Reviewer #2: This manuscript has been improved a lot after revision. Some comments are given below.

1. In line 467, it is said that “We obtained 30% subsamples of cells from each cluster (16,351 cells in total ).” However, in the response for referee#2-2, it is said that “The total number of cells after subsampling was 10,324. Genes with fewer than 10 cells expressed in the subsampled state were filtered out, and 16,351 genes were used as variables.” Please double check.

We apologize for our error.

The number of cells before subsample was incorrect. The correct number is 17730 cells, not 16,351 cells. The text has been corrected as follows.

“We obtained 30% subsamples of cells from each cluster (10,324 cells were extracted from 17,730 cells in total)” (page 25, line 471)

In addition to this, an error related to this has been also corrected as follows.

“As a result of applying this criterion, the number of DEGs was 7495, which was nearly half of the total (42%) of 16,351 mouse genes” (page 14, line 229).

2. For UpSet plots (Fig. S1b&c), results are confusing. For example, in Fig. S1b, the number of interaction features of all four methods is 32, but the number of interaction features of SCAD and Lasso is 27<32. Please check.

The numbers are correct, but we understand that the meaning of UpSet plot is somewhat confusing for readers. In our case (default setting of UpSet plot), the second column of Fig. S1b shows the number of genes extracted by all four methods. The third column shows the number of genes that are shared only between SCAD and Lasso. The other columns are similarly defined by sharing genes only between the marked methods.

To avoid confusion, we have added the following text to the figure legend.

“Each columns represents number of genes that are shared by only the marked sets.” (page 34, line 655)
